# Resource limitation and responses to rivals in males of the fruit fly *Drosophila melanogaster*

**DOI:** 10.1111/jeb.12924

**Published:** 2016-07-15

**Authors:** J. S. Mason, W. G. Rostant, T. Chapman

**Affiliations:** ^1^ School of Biological SciencesUniversity of East AngliaNorwichUK

**Keywords:** carbohydrate, diet, mating duration, mating latency, nutrition, protein, reproduction

## Abstract

Diet has a profound direct and indirect effect on reproductive success in both sexes. Variation in diet quality and quantity can significantly alter the capacity of females to lay eggs and of males to deliver courtship. Here, we tested the effect of dietary resource limitation on the ability of male *Drosophila melanogaster* to respond adaptively to rivals by extending their mating duration. Previous work carried out under *ad libitum* diet conditions showed that males exposed to rivals prior to mating significantly extend mating duration, transfer more ejaculate proteins and achieve higher reproductive success. Such adaptive responses are predicted to occur because male ejaculate production may be limited. Hence, ejaculate resources require allocation across different reproductive bouts, to balance current vs. future reproductive success. However, when males suffer dietary limitation, and potentially have fewer reproductive resources to apportion, we expect adaptive allocation of responses to rivals to be minimized. We tested this prediction and found that males held on agar‐only diets for 5–7 days lost the ability to extend mating following exposure to rivals. Interestingly, extended mating was retained in males held on low yeast/sugar: no sugar/yeast diet treatments, but was mostly lost when males were maintained on ‘imbalanced’ diets in which there was high yeast: no sugar and vice versa. Overall, the results show that males exhibit adaptive responses to rivals according to the degree of dietary resource limitation and to the ratio of individual diet components.

## Introduction

Quantitative and qualitative variations in nutrition significantly influence reproductive physiology and behaviour as well as overall reproductive success (Thompson, 1999). Specific dietary components are essential for normal metabolism and development (House, 1962), and an extensive body of research in invertebrates demonstrates the significant effects of diet on reproduction in both sexes (e.g. Chippindale *et al*., 1993, 1997; Chapman & Partridge, 1996; Engqvist & Sauer, 2003; Carey *et al*., 2008; Fricke *et al*., 2008, 2015; Maklakov *et al*., 2008; Perry & Rowe, 2010; Perez‐Staples *et al*., 2011; Lewis *et al*., 2012; Taylor *et al*., 2013; Hopwood *et al*., 2014).

In male *Drosophila melanogaster*, an optimal level of nutrition is required to maximize reproductive success through the initiation of effective post‐mating responses in females (Fricke *et al*., 2008). Diet can also have direct effects on a male's ability to mate and to transfer sperm. For example, male medflies (*Ceratitis capitata*) fed a protein‐deprived diet mate at a lower frequency than those fed on higher protein diets, but transfer more sperm during mating (Blay & Yuval, 1997). However, the same study found that females paired with protein‐deprived males are quicker to re‐mate, resulting in reduced reproductive success and indicating diet is a major factor in determining a male's reproductive success. Diet quality can also influence important reproductive characteristics such as pheromone production and blend (e.g. in the Caribbean fruit fly *Anastrepha suspensa;* Sivinski & Heath, 1988) and has wide‐ranging effects on reproductive traits in general (Aluja *et al*., 2001; Perez‐Staples *et al*., 2008, 2011; Taylor *et al*., 2013).

Diet is expected to influence both male reproductive success and female reproductive success via fitness trade‐offs (Parker & Pizzari, 2010). The idea is that a reduction in dietary resources leads males to reallocate resources to survival at the expense of reproductive processes, thereby limiting the options for optimal allocation of resources to reproduction. The frequent reports of evolutionary and proximate trade‐offs between reproductive traits and survival across many species (e.g. Holehan & Merry, 1985; Partridge & Harvey, 1988; Partridge & Sibly, 1991; Stearns, 1992; Partridge *et al*., 1999; Flatt, 2011) suggest that such mechanisms could be common. Significant progress has been made towards understanding the mechanistic basis of such trade‐offs through investigations of the effect of diet on reproduction and survival (e.g. Piper *et al*., 2005a, b; Partridge & Gems, 2006; Libert *et al*., 2007; Grandison *et al*., 2009; Partridge *et al*., 2011; Wigby *et al*., 2011; Tatar *et al*., 2014). For example, it is well established that female *D. melanogaster* reared on high‐quality diets mate more and are more fecund, but have a shortened lifespan compared to those reared on low‐quality diets (e.g. Chippindale *et al*., 1993, 1997; Chapman & Partridge, 1996). The role of nutrient signalling genes in the insulin signalling and target of rapamycin pathways in mediating the relationship between nutrition, reproduction and lifespan is now well established (e.g. Partridge *et al*., 2011; Partridge *et al*., 2005; Grandison *et al*., 2009; Tatar *et al*., 2014).

This extensive body of work suggests that nutritional state is likely to influence many if not all aspects of the reproductive repertoire of both sexes. In this study, we focussed on males and investigated the effect of dietary resource availability in a novel context – namely on the ability of male *D. melanogaster* to express adaptive responses to rivals. Crucial to a male's overall reproductive success, in addition to the ability to mate and transfer sperm, is his ability to respond adaptively to the presence of rival males (Bretman *et al*., 2011a, b). For example, a male Norway rat (*Rattus norvegicus)* will ejaculate more sperm in the presence of another male (Pound & Gage, 2004) and gain higher fertilization success by doing so. In an environment in which there is sperm competition, sperm transfer to the female is also observed to increase in response to the presence of rival males (e.g. in the crickets *Acheta domesticus* and *Gryllodes supplicans*, Gage & Barnard, 1996). Males of the South American Fruit fly *Anastrepha fraterculus* are also reported to respond plastically to perceived levels of immediate sperm competition intensity as indicated by the number of rival males (Abraham *et al*., 2015). Similarly, Bretman *et al*. (2009) used *D. melanogaster* to investigate male responses to rivals and found that mating duration and paternity increased significantly following exposure of males to their rivals prior to mating. The extension of mating duration, in this context, is largely controlled by males (Bretman *et al*., 2013b). The evidence suggests that this trait represents an adaptive strategy by males to increase paternity under competitive conditions (Bretman *et al*., 2009). Although the absolute number of rivals had little effect on mating duration, an increased length of exposure to rivals prolonged subsequent mating duration (Bretman *et al*., 2010).

Although the responses of males to rivals are known to be important in determining a male's fitness (Bretman *et al*., 2009, 2013a), nothing is yet known about how these responses are influenced by diet. The existence of plastic responses to rivals suggests that extended mating and/or ejaculate production is costly. That extended mating is linked to ejaculate production is shown by the finding that males that exhibited extended matings following exposure to rivals transferred more of two seminal fluid proteins measured (Wigby *et al*., 2009). Hence, extended mating and ejaculate production may be limiting, which may select for the evolution of mechanisms to adaptively allocate between current and future reproductive bouts. This predicts that the degree of plasticity in ejaculate allocation should be affected by the availability of reproductive investment/ejaculate to allocate and the probability of future reproductive opportunities (Tazzyman *et al*., 2009; Parker & Pizzari, 2010). Ejaculate‐mediated post‐mating responses elicited in females by males show a significant diminution with decreasing diet quality (Fricke *et al*., 2008). Hence, variation in nutrition provides a mechanism by which to experimentally alter reproductive investment in ejaculates.

Should an individual become resource limited because of a poor diet, we would expect the expression of adaptive allocation decisions to be minimized or absent. In this study, we tested this prediction. We asked whether males experiencing a reduced quantity or quality of diet lost the ability to respond adaptively to rivals. Diet quantity was investigated by comparing full diets to those containing only agar, and diet quality using diets that contained only yeast or sucrose components. Four separate experiments were conducted. In the first, we varied yeast level against a zero sucrose background and compared this to the standard and agar‐only diets. In the second, we did the reverse. In the third, we conducted a reciprocal design in which the presence or absence of the two major diet components against a background full diet was tested. In the final experiment, we increased the replication and tested the repeatability of all treatments from the first three experiments in a simultaneous, reciprocal design. In addition, we counted a sample of all the offspring produced by females mated to males from each of the treatments.

## Materials and methods

### General

All flies were taken from the Dahomey wild‐type population described in our earlier, related studies (e.g. Bretman *et al*., 2009, 2010, 2011a, b). The wild‐type stock was maintained at 25 °C on a 12 : 12 light : dark cycle in overlapping generation cage cultures. The standard sucrose–yeast (SY) food comprised 100 g brewer's yeast, 50 g sucrose, 15 g agar, 30 mL Nipagin (10% w/v solution), 3 mL propionic acid, 1 L water. All experiments were conducted at 25 °C and in a humidified constant temperature room (~ 50% RH), using glass vials (75 mm height × 25 mm diameter) containing 7 mL of SY food. To collect adults for the experiments, wild‐type females were allowed to oviposit on agar–grape juice plates (50 g agar, 600 mL red grape juice, 42.5 mL Nipagin (10% w/v solution), 1.1 L water) to which a drop of yeast paste was added. First‐instar larvae were collected the following day and groups of 100 transferred to vials containing SY medium. Vials were incubated at standard conditions for 10 days during larval development. Virgin adults were ice‐anaesthetized upon eclosion for sexing.

### Experiment 1. The effect of variation in dietary yeast against zero sucrose on male responses to rivals after 7 days of diet exposure

We tested whether there were differences in the extent of male responses to rivals when the major components of their diet were manipulated. In this first experiment, yeast was varied against zero sucrose content vs. the normal and agar‐only diets. Males and females were collected as virgins at eclosion using ice anaesthesia. Females were housed in groups of five per vial on the normal SY diet, supplemented with added yeast granules until use in the experiment. Males were collected in groups of 10 per vial and housed on a diet of normal SY for 2 days post‐eclosion to reach sexual maturity (Eastwood & Burnet, 1977) and allow their reproductive systems to fully develop and therefore to express any potential allocation responses. Males were then randomly allocated to one of four different diet treatments to test the effect of varying protein (yeast) concentration in the absence of a carbohydrate (sucrose) source:

1. standard control diet (100% yeast: 100% sucrose)
2. standard yeast, no sucrose (100% yeast: 0% sucrose)
3. low yeast, no sucrose (20% yeast: 0% sucrose)
4. agar only (0% yeast: 0% sucrose).

Males were placed in the four food treatment groups in vials either singly or with three rivals for 7 days until the mating test. All males were transferred to fresh agar‐only vials for mating to prevent any immediate responses of males held on poor diets prior to mating to a better quality diet. For the single male (no rival) treatments, the single male in each vial was used in the mating tests. For the exposure to rival treatments, one male of the four housed together was randomly chosen for the mating tests. Pairs were aspirated into each agar‐only mating vial. Flies that did not mate within 3 h were discarded. Copulations that lasted < 5 min do not transfer sperm (Gilchrist & Partridge, 2000) and were excluded. Copulations of > 45 min were also excluded from the data and represent rare occurrences where individuals failed to separate following mating. The introduction time and start and finish of matings were recorded to the nearest minute. Forty males were initially allocated to each treatment, with the final sample sizes at the time of the mating tests being (for no rivals and then rivals treatments, respectively): 100% yeast: 100% sucrose, *n* = 40, 36; 100% yeast: 0% sucrose, *n* = 34, 37; 20% yeast: 0% sucrose, *n* = 33, 33; 0% yeast: 0% sucrose, *n* = 18, 8. The overall percentage of males surviving from the initial set‐up to the mating tests across rival and no rival treatments was therefore 95%, 89%, 83% and 33% (100% yeast: 100% sucrose; 100% yeast: 0% sucrose; 20% yeast: 0% sucrose; 0% yeast: 0% sucrose diets, respectively). The agar‐only diet therefore exerted severe nutritional stress.

### Experiment 2. The effect of variation in dietary sucrose against zero yeast on male responses to rivals after 7 days of diet exposure

The second experiment was conducted exactly as described above, except that males were placed in one of four nutritional treatments to test the effect of varying carbohydrate (sucrose) concentration in the absence of a protein (yeast) source. The diets were as follows:

1. standard control diet (100% yeast: 100% sucrose)
2. no yeast, standard sucrose (0% yeast: 100% sucrose)
3. no yeast, low sucrose (0% yeast: 20% sucrose)
4. agar only (0% yeast: 0% sucrose).

Males were allocated randomly to one of the 4 food treatment groups and placed in vials either singly or with rivals for 7 days until mating. Mating tests were then conducted as described above. 40 males were initially allocated to each treatment, final sample sizes for the mating tests were (no rivals and then rivals treatments, respectively): 100% yeast: 100% sucrose, *n* = 27, 26; 0% yeast: 100% sucrose, *n* = 30, 26; 0% yeast: 20% sucrose, *n* = 16, 18; 0% yeast: 0% sucrose *n* = 17, 20. The overall percentage of males surviving to the mating tests (rival and no rival treatments) was 66%, 70%, 42% and 46% (100% yeast: 100% sucrose; 0% yeast: 100% sucrose; 0% yeast: 20% sucrose; 0% yeast: 0% sucrose diets, respectively). As above, the agar‐only diet imposed severe nutritional stress.

### Experiment 3. The effect of removal of dietary yeast and/or sucrose on male responses to rivals after 5 days of diet exposure

In the third experiment, a fully reciprocal test of diet components was conducted, in which the effect of removing either yeast or sucrose was determined. Males were allocated randomly to one of four dietary treatments:

1. standard control diet (100% yeast: 100% sucrose)
2. no yeast, standard sucrose (0% yeast: 100% sucrose)
3. standard yeast, no sucrose (100% yeast: 0% sucrose)
4. agar only (0% yeast: 0% sucrose).

Mortality had been high in the first two experiments for males held on the agar‐only diet for 7 days (with only ~ 33% of males surviving to the mating tests). We wanted to reduce this mortality in experiment 3 to allow a more balanced experimental design and increase the sample size for the agar‐only diet treatment. Hence, we held males on their respective diets for 5 days prior to mating tests. Forty males were initially allocated to each treatment, and final sample sizes for the mating tests were as follows (no rivals and then rivals treatments, respectively): 100% yeast: 100% sucrose, *n* = 39, 39; 0% yeast: 100% sucrose, *n* = 38, 34; 100% yeast: 0% sucrose, *n* = 36, 38; 0% yeast: 0% sucrose *n* = 37, 36. The overall percentage of males surviving to the mating tests (rival and no rival treatments) was 97%, 90%, 93% and 91% (100% yeast: 100% sucrose; 0% yeast: 100% sucrose; 100% yeast: 0% sucrose; 0% yeast: 0% sucrose diets, respectively). The decreased exposure to agar‐only diets from 7 to 5 days therefore increased survival from 33% to > 90%.

### Experiment 4. The effect of reciprocal variation in dietary yeast and sucrose on male responses to rivals

In this final experiment, we increased the level of replication and tested for repeatability of all the previous experimental diet treatments by placing males on all six diets simultaneously and assaying their responses to rivals. Therefore, the diet treatments were as follows:

1. standard control diet (100% yeast: 100% sucrose)
2. standard yeast, no sucrose (100% yeast: 0% sucrose)
3. no yeast, standard sucrose (0% yeast: 100% sucrose)
4. low yeast, no sucrose (20% yeast: 0% sucrose)
5. no yeast, low sucrose (0% yeast: 20% sucrose)
6. agar only (0% yeast: 0% sucrose)

Prior to conducting the tests of rival responses in males held on these diets, we tested the survival responses of males to the different diet treatments. We did this in order to: (i) test whether there was any survival difference between males kept on their own vs. with rivals, and (ii) gauge the optimal age for the mating tests, that is the age at which the survival of nutritionally stressed cohorts started to decline steeply. We placed wild‐type males reared under standard density conditions as before and placed 100 males each in treatments of one per vial (‘no rivals’ males) or four per vial (‘rivals’ males) on each of the above diets, from eclosion onwards. We then checked survival daily until the experiment was terminated at day 33. In the ‘rivals’ treatments, dead males were removed daily and numbers per vial were kept constant by consolidating survivors at 4/vial. From this experiment, we chose 6 days post‐eclosion as the most appropriate age for testing the responses of males to rivals (see Results).

For the main experiment, males were reared exactly as above and allocated randomly to one of the six diet treatments and placed in vials either singly or with rivals for 6 days until the mating tests, which we conducted as described above. Sixty males were allocated to each treatment combination on the day of mating. Final sample sizes for the numbers of (‘no rivals’, ‘rivals’) males that mated within the 3 h window were as follows: 100% yeast: 100% sucrose = 59, 60; 100% yeast: 0% sucrose = 59, 56; 0% yeast: 100% sucrose = 56, 57; 20% yeast: 0% sucrose = 55, 58; 0% yeast: 20% sucrose = 46, 53; 0% yeast: 0% sucrose = 54, 58. Each male that mated was subsequently monitored daily for survival, to determine starvation resistance. The mated females were placed individually into SY vials for 24 h. The total number of offspring from each of these vials was counted 12 days later.

### Statistical analysis

Statistical analyses were performed using R v 3.1.2 (R Core Team, 2014). General linear models (GLMs) with normal errors were used, and significance of factors was determined by stepwise model reduction from the maximal model via likelihood ratio tests whereby the deviance (*D*)(−2 times the difference between the log‐likelihood of the reduced model and the log‐likelihood of the full model) was tested for significance by comparison with an *F* distribution. Maximal models included the factors diet (four or six levels in each experiment) and rival (two levels: rivals/no rivals) along with an interaction term (diet × rival), and the significance of main effects was tested after removal of the interaction term. Response variables included mating latency, which was log‐transformed to improve normality, and mating duration. Offspring counts from Experiment 4 were analysed in a similar manner using a zero‐inflated negative binomial GLM. Planned contrasts between rivals and no rivals within each diet treatment were also performed using the ‘glht()’ function in the ‘multcomp’ package (Hothorn *et al*., 2008).

## Results

### Experiment 1. The effect of variation in dietary yeast against zero sucrose on male responses to rivals after 7 days of diet exposure

**Figure 1:**
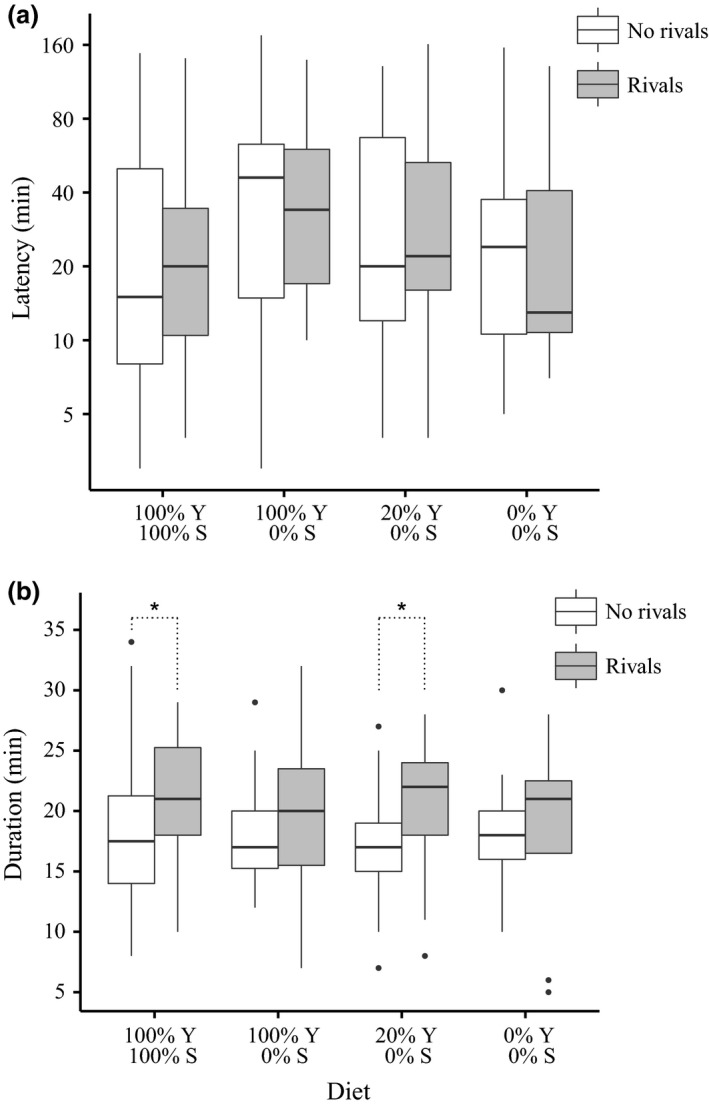
Experiment 1: Mating responses to rivals under varying yeast (Y) with sucrose (S) held at zero. (a) Boxplots of mating latency (plotted on logarithmic scale) as a function of diet and male rival presence. Final latency sample sizes for ‘no rivals’, ‘rivals’ males were 100% Y: 100% S = 37, 36; 100% Y: 0% S = 35, 37; 20% Y: 0% S = 33, 33; 0% Y: 0% S = 19, 8. (b) Boxplots of mating duration as a function of diet and male rival presence. Final duration sample sizes for ‘no rivals’, ‘rivals’ males were 100% Y: 100% S = 36, 36; 100% Y: 0% S = 34, 35; 20% Y: 0% S = 33, 33; 0% Y: 0% S = 18, 8. Median represented by horizontal line within box, with box representing the interquartile range (IQR) and whiskers the highest/lowest value within 1.5 * IQR. Outliers represented by points. Significant planned contrasts: *P <* *0.05.

Mating latency was significantly affected by diet (GLM, *F*_3,234_ = 4.333, *P *=* *0.005, Fig. 1a). There was no significant interaction effect (GLM, *F*_3,230_ = 0.048, *P *=* *0.986), nor any main effect of rivals (GLM, *F*_1,233_ = 0.058, *P *=* *0.809) on mating latency. *Post hoc* Tukey's HSD tests revealed that latency was significantly longer (*P *<* *0.05) for males held on the 100% yeast: 0% sucrose diet than for males held on the full diet (100% yeast: 100% sucrose). All other diet comparisons were nonsignificant. Five outliers were removed from the analysis of mating duration (< 5 min or > 45 min; namely one ‘no rivals’ and two ‘rivals’ males from the 100% yeast: 0% sucrose diet, and one ‘no rivals’ male each from the 100% yeast: 100% sucrose and 0% yeast: 0% sucrose diets). As expected, based on previous work (Bretman *et al*., 2009), males exposed to rivals prior to mating mated for significantly longer (GLM, *F*_1,231_ = 13.594, *P *<* *0.001, Fig. 1b). However, the planned contrast analyses within diet treatments revealed that this effect was not apparent for males held on the 100% yeast: 0% sucrose (*z *=* *0.966, *P *=* *0.803) or the 0% yeast: 0% sucrose, (*z *=* *0.196, *P *=* *0.999) diets. There was no significant interaction effect (GLM, *F*_3,225_ = 1.060, *P *=* *0.367) or main effect of diet (GLM, *F*_3,228_ = 1.226, *P *=* *0.301) on mating duration.

### Experiment 2. The effect of variation in dietary sucrose against zero yeast on male responses to rivals after 7 days of diet exposure

**Figure 2:**
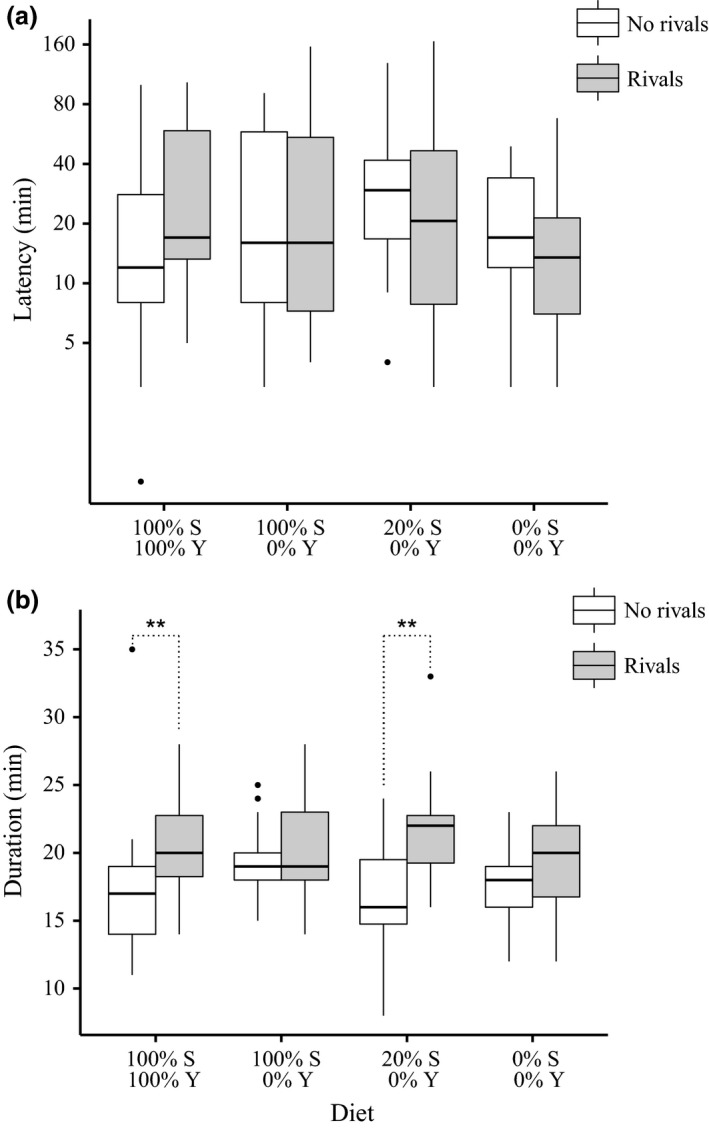
Experiment 2: Mating responses to rivals under varying sucrose (S) with yeast (Y) held at zero. (a) Boxplots of mating latency (plotted on logarithmic scale) as a function of diet and male rival presence. Final latency sample sizes for ‘no rivals’, ‘rivals’ males were 100% S: 100% Y = 25, 26; 100% S: 0% Y = 25, 26; 20% S: 0% Y = 16, 18; 0% S: 0% Y = 17, 20. (b) Boxplots of mating duration as a function of diet and male rival presence. Final duration sample sizes for ‘no rivals’, ‘rivals’ males were 100% S: 100% Y = 25, 26; 100% S: 0% Y = 25, 26; 20% S: 0% Y = 16, 18; 0% S: 0% Y = 16, 20. Boxplots as in Fig. 1. Significant planned contrasts: *P <* *0.05, **P <* *0.01.

Consistent with above, there was again no significant interaction effect (GLM, *F*_3,165_ = 1.346, *P* = 0.261), nor any main effect of rivals (GLM, *F*_1,171_ = 0.401, *P* = 0.527) or diet (GLM, *F*_3,169_ = 1.161, *P* = 0.326) on mating latency (Fig. 2a). One outlier (a ‘no rivals’ male from the 0% sucrose: 0% yeast diet) was removed from the analysis of mating duration (< 5 min). Consistent with Experiment 1, there was a significant main effect of rivals on mating duration, with males exposed to rivals prior to mating having significantly longer mating durations overall (GLM, *F*_1,170_ = 18.928, *P *<* *0.001, Fig. 2b). This effect was observed for males on the 100% sucrose: 100% yeast (planned contrasts, *z* = 3.271, *P* = 0.004) and 20% sucrose: 0% yeast (*z* = 3.651, *P* = 0.001) diets. However, males exposed to rivals on the 100% sucrose: 0% yeast (*z* = 0.804, *P* = 0.888) and the 0% sucrose: 0% yeast (*z* = 1.274, *P* = 0.596) diets showed no difference in mating duration in comparison with males housed alone. There was no significant interaction effect (GLM, *F*_3,164_ = 2.236, *P* = 0.086) or main effect of diet (GLM, *F*_3,167_ = 0.877, *P* = 0.454) on mating duration.

### Experiment 3. The effect of removal of dietary yeast and/or sucrose on male responses to rivals after 5 days of diet exposure

**Figure 3:**
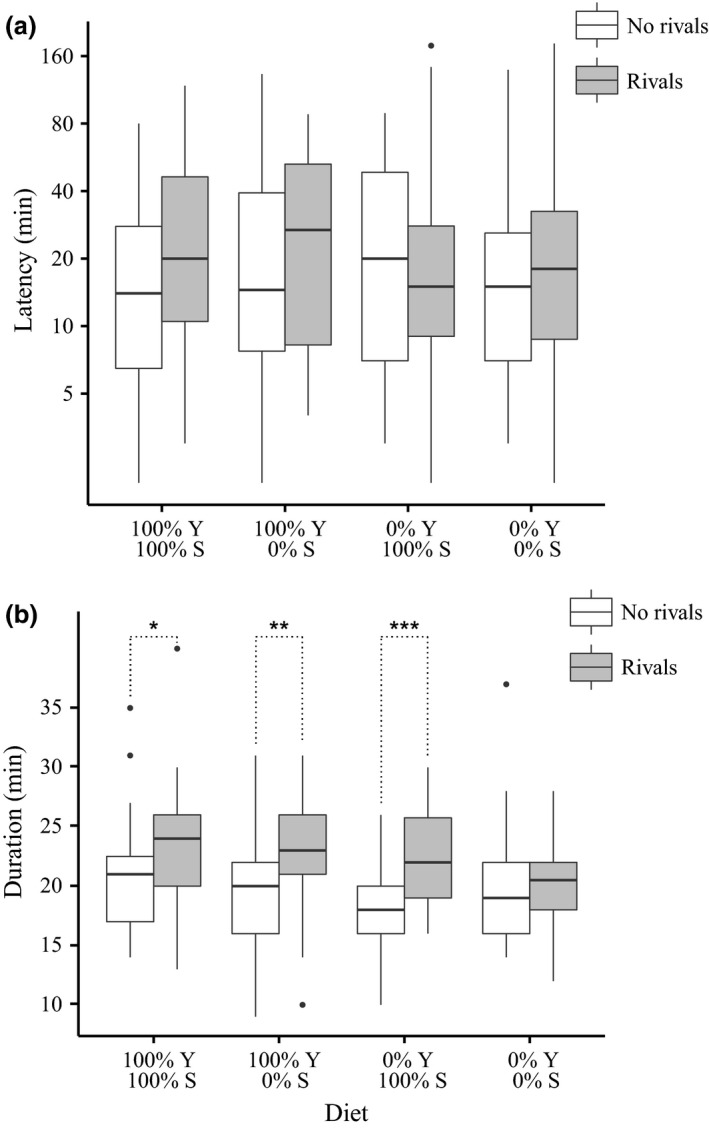
Experiment 3: Mating responses to rivals under reciprocal variation of yeast (Y) and sucrose (S). (a) Boxplots of mating latency (plotted on logarithmic scale) as a function of diet and male rival presence. Final latency sample sizes for ‘no rivals’, ‘rivals’ males were 100% Y: 100% S = 39, 39; 100% Y: 0% S = 36, 38; 0% Y: 100% S = 38, 34; 0% Y: 0% S = 37, 36. (b) Boxplots of mating duration as a function of diet and male rival presence. Final duration sample sizes for ‘no rivals’, ‘rivals’ males were 100% Y: 100% S = 39, 39; 100% Y: 0% S = 36, 38; 0% Y: 100% S = 38, 34; 0% Y: 0% S = 37, 36. Boxplots as in Fig. 1. Significant planned contrasts: *P <* *0.05, **P <* *0.01, ***P <* *0.001.

In the third experiment, the yeast and sucrose components of the diet were both varied against standard levels of other diet components. We saw no significant interaction effect (GLM, *F*_3,289_ = 1.063, *P* = 0.365), nor any main effect of diet (GLM, *F*_3,293_ = 0.314, *P* = 0.815) or rivals (GLM, *F*_1,295_ = 1.272, *P* = 0.260) on mating latency (Fig. 3a). We again observed a significant interaction effect of diet treatment on mating duration (GLM, *F*_3,289_ = 3.017, *P* = 0.030) and that males held on the 0% sucrose: 0% yeast agar‐only diet did not respond to the presence of rivals by increasing mating duration (planned contrasts, *z* = 0.085, *P* = 1.000). After removal of the interaction term, both diet (GLM, *F*_3,292_ = 2.767, *P* = 0.042) and rivals (GLM, *F*_1,292_ = 25.405, *P *<* *0.001) had significant main effects on mating duration (Fig. 3b). This experiment was the only one in which we observed extended mating in response to rivals in males held on 100%: 0% diets.

### Experiment 4. The effect of reciprocal variation in dietary yeast and sucrose on male responses to rivals

Daily monitoring of the test cohorts prior to the main experiment showed > 95% survival across all treatments for the first 6 days post‐eclosion and a dramatic increase in mortality for the 0% yeast: 20% sucrose and 0% yeast: 0% sucrose diets immediately thereafter (Fig. S1). This suggested that males held for 6 days on these two diets were experiencing physiological stress as a precursor to increased mortality. However, there was no significant effect of the presence or absence of rivals on male survival during this 6‐day period (Fig. S1). Hence, we chose day 6 for the mating tests to represent a period of diet exposure that was likely to exert physiological effects on males, but which were not yet manifested as lifespan differences.

**Figure 4:**
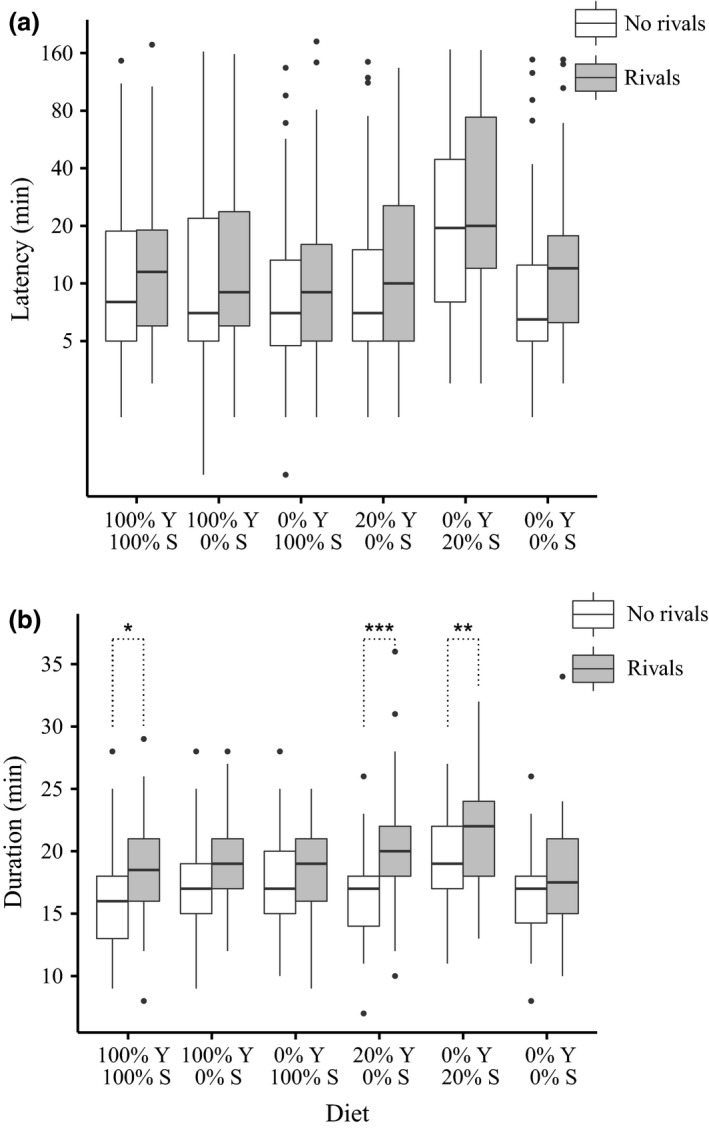
Experiment 4: Mating responses to rivals under reciprocal variation of yeast (Y) and sucrose (S). Mating tests conducted after 6 days of exposure to all six diet treatments. (a) Boxplots of mating latency (plotted on logarithmic scale) as a function of diet and male rival presence. Final latency sample sizes for ‘no rivals’, ‘rivals’ males were 100% Y: 100% S = 59, 60; 100% Y: 0% S = 59, 56; 0% Y: 100% S = 56, 57; 20% Y: 0% S = 55, 58; 0% Y: 20% S = 46, 53; 0% Y: 0% S = 54, 58. (b) Boxplots of mating duration as a function of diet and male rival presence. Final duration sample sizes for ‘no rivals’, ‘rivals’ males were 100% Y: 100% S = 59, 60; 100% Y: 0% S = 59, 56; 0% Y: 100% S = 56, 57; 20% Y: 0% S = 55, 58; 0% Y: 20% S = 46, 53; 0% Y: 0% S = 54, 58. Boxplots as in Fig. 1. Significant planned contrasts: *P <* *0.05, **P <* *0.01, ***P <* *0.001.

There was no significant interaction effect (GLM, *F*_5,659_ = 0.390, *P* = 0.856), but both diet (GLM, *F*_5,664_ = 10.439, *P *<* *0.001) and rivals (GLM, *F*_1,664_ = 6.807, *P* = 0.009) had significant main effects on mating latency (Fig. 4a). However correcting for multiple testing, using planned contrasts as before, we failed to find any significant differences in mating latency between ‘rivals’ and ‘no rivals’ males in any of the individual diet treatments. As in Experiment 3, we found a significant interaction (rival × diet) effect on mating duration (GLM, *F*_5,659_ = 3.106, *P* = 0.009). Planned contrasts revealed that, consistent with above, it was males on the 0% sucrose: 0% yeast agar‐only diet diets that failed to respond to the presence of rivals by increasing mating duration (*z* = 1.040, *P* = 0.880). Males on the full (100% yeast: 100% sucrose) (*z* = 3.071, *P* = 0.013), 20% yeast: 0% sucrose (*z* = 5.741, *P *<* *0.001) and 0% yeast: 20% sucrose (*z* = 3.196, *P* = 0.008) diets all showed a significant mating duration response to rivals. After removal of the interaction term, both diet (GLM, *F*_5,664_ = 10.283, *P *<* *0.001) and rivals (GLM, *F*_1,664_ = 44.818, *P *<* *0.001) had significant main effects on mating duration (Fig. 4b).

Analysis of offspring counts from all Experiment 4 matings revealed that there was no significant effect of the diet treatments (Fig. S2a). However, post‐mating starvation resistance was significantly affected by the interaction of diet and rival (GLM, *F*_5,649_ = 7.199, *P *<* *0.001). After removal of the interaction term, both diet (GLM, *F*_5,654_ = 167.429, *P *<* *0.001) and rivals (GLM, *F*_1,654_ = 13.457, *P *<* *0.001) had significant main effects on number of days survived until starvation (Fig. S2b). Planned contrast analyses of starvation resistance within diet treatments revealed a rival effect for males held only on the standard control (*z* = 2.828, *P* = 0.028) and 100% yeast: 0% sucrose (*z* = 6.103, *P *<* *0.001) diets.

The results of all the analyses for mating duration across all four experiments are summarised in Tables 1 and 2.

**Table 1:** Summary of general linear model analyses of deviance of mating duration for males held on different diets and exposed to rivals or no rivals in all four experiments. Significant results in bold

Effect	Exp 1 (Yeast varied/no sucrose)	Exp 2 (Sucrose varied/no yeast)	Exp 3 (Removal of yeast and/or sucrose)	Exp 4 (Reciprocal variation of yeast and sucrose)
Diet	*F*_3,228_ = 1.226 *P *=* *0.301	*F*_3,167_ = 0.877 *P* = 0.454	***F***_**3,292**_ ** = 2.767** ***P*** = **0.042**	***F***_**5,664**_ ** =** **10.283** ***P *** **<** *** *** **0.001**
Rival	***F***_**1,231**_ ** = 13.594** ***P *** **<** *** *** **0.001**	***F***_**1,170**_ ** = 18.928** ***P *** **<** *** *** **0.001**	***F***_**1,292**_ ** = 25.405** ***P *** **<** *** *** **0.001**	***F***_**1,664**_ ** =** **44.818** ***P *** **<** *** *** **0.001**
Diet × Rival	*F*_3,225_ = 1.060 *P* = 0.367	*F*_3,164_ = 2.236 *P* = 0.086	***F***_**3,289**_ ** = 3.017** ***P*** = **0.030**	***F***_**5,659**_ ** = 3.106** ***P *** **=** *** *** **0.009**

**Table 2:** Summary results of the planned contrasts of mating duration response between ‘rivals’ and ‘no rivals’ treatments within each diet treatment. Significant results in bold

Contrast	Exp 1 (Yeast varied/no sucrose)	Exp 2 (Sucrose varied/no yeast)	Exp 3 (Removal of yeast and/or sucrose)	Exp 4 (Reciprocal variation of yeast & sucrose)
100% yeast, 100% sucrose: rivals vs. no rivals	***z *** **=** *** *** **2.592** ***P *** **=** *** *** **0.038**	***z *** **=** *** *** **3.271** ***P *** **=** *** *** **0.004**	***z *** **=** *** *** **2.878** ***P *** **=** *** *** **0.016**	***z *** **=** *** *** **3.071** ***P *** **=** *** *** **0.013**
0% yeast, 0% sucrose: rivals vs. no rivals	*z *=* *0.196 *P *=* *0.999	*z *=* *1.274 *P *=* *0.596	*z *=* *0.085 *P *=* *1.000	*z *=* *1.040 *P *=* *0.880
100% yeast, 0% sugar: rivals vs. no rivals	*z *=* *0.966 *P *=* *0.803		***z *** **=** *** *** **3.055** ***P *** **=** *** *** **0.009**	*z *=* *2.538 *P *=* *0.065
20% yeast, 0% sucrose: rivals vs. no rivals	***z *** **=** *** *** **2.921** ***P *** **=** *** *** **0.014**			***z *** **=** *** *** **5.741** ***P *** **<** *** *** **0.001**
0% yeast, 20% sucrose: rivals vs. no rivals		***z *** **=** *** *** **3.651** ***P *** **=** *** *** **0.001**		***z *** **=** *** *** **3.196** ***P *** **=** *** *** **0.008**
0% yeast, 100% sugar: rivals vs. no rivals		*z *=* *0.804 *P *=* *0.888	***z *** **=** *** *** **4.166** ***P *** **<** *** *** **0.001**	*z *=* *0.964 *P *=* *0.914

## Discussion

Across the four experiments, there were no consistent effects of either diet or the presence of rivals on a male's latency to mating. This is in line with the results of previous studies (Bretman *et al*., 2009, 2010, 2011a, b). In contrast, the results for the effect of diet and the presence of rivals on mating duration were highly repeatable (Tables 1 and 2). The results of all four experiments showed that males held on an agar‐only diet for 5–7 days prior to mating never responded to the presence of rivals by extending mating duration, whereas males held on the normal diets did. The results across the four experiments were therefore consistent in showing that males held under strong resource limitation (agar‐only) for 5–7 days prior to mating lost the ability to respond to rivals. Interestingly, males held on highly ‘imbalanced’ diets of either 100% yeast: 0% sugar or 0% yeast: 100% sugar also showed no extended mating duration in response to rivals in three of four experiments tested. In contrast, males held on the 20% yeast: 0% sugar or 0% yeast: 20% sugar diets significantly extended mating in response to rivals in all four experiments. This suggests that it is partly the balance or ratio of major nutrient components (yeast and sugar), but not their overall level, that constitutes the primary determinant of whether males can respond adaptively to rivals.

Males held on the agar‐only diets were under severe resource limitation, as shown by their elevated mortality rate following the timing of the mating tests, in comparison with males held on the other diets. These males are assumed to have had fewer resources to allocate across different reproductive episodes. Therefore, we predicted that such males would show limited adaptive responses to rivals. The findings were consistent with this prediction. The agar‐only diet males were unable to express altered mating duration in response to rivals, presumably because they had no resources to strategically allocate to current vs. future mating opportunities, or that the resources they had were traded off against the need to maintain survival for long enough to reproduce. The observation that males were near to the point at which survival would decline steeply at the time of the mating tests is consistent with the idea that on the agar‐only diet there were few resources to allocate to different reproductive processes. If males under starvation conditions were investing maximally in what might constitute their final mating, we might also expect to see those males mating for longer overall; however, there was no such signal in our data. A male's reproductive success depends critically on his ability to respond adaptively to rival males, and prolonged copulation enhances male fitness by increasing success in sperm competition and by decreasing a mate's receptiveness to rival males (Bretman *et al*., 2010). The loss of the ability to respond to rivals under severe diet limitation is a novel result and suggests that the pay‐offs of behavioural plasticity in this context interact with resource availability.

Perhaps the most interesting result was that a low level of either yeast or sugar (in the absence of the other major diet component) consistently triggered males to respond to rivals via significantly extending mating duration. Both types of food component may therefore feed into the pathway required for the expression of such responses or there may be some interconversion of nutrients between different pathways under diet‐limiting environments (Sinclair *et al*., 2011). However, males held on diets containing a high level of yeast or sugar (in the absence of the other major diet component) were not able (in three of four experiments tested) to significantly extend mating in response to rivals. The underlying explanation is not yet known, but it is possible that a very high level of one diet component in the absence of the other is more indicative to males of nutritional limitation than is the case for a lower ratio of the same components. It is also possible that imbalanced diets are more toxic to males and thus induce higher stress leading to the lack of mating duration/ejaculate allocation. Future tests will be useful here, including the possibility that the 100%: 0% diets induce detoxification pathways, potentially leading to greater potential trade‐offs with reproductive allocation. The result suggests that any models developed to explore the underlying balance of costs and benefits involved in responding to rivals need not only to explore whether there are resource thresholds for the expression of adaptive responses but also to capture the additional complexity of the ratio of diet components.

The data from the final experiment showed that there were no significant differences in male survival across different diets up to the point at which the mating tests were conducted (Fig. S1). There was also no effect of rivals on male survival up to the time of the mating tests on any of the diets. Therefore, we have no evidence that differences in competition between males, or differential effects arising from interactions of male survival with diet confounded the effects observed. Following the mating tests in the final experiment, we measured the starvation resistance of all males and observed significant differences due to the interaction of diet and rivals. Males exposed to rivals had lower post‐mating starvation survival than their counterparts not so exposed on the standard control diet and the 100% yeast: 0% sugar diet. On all other diets, there was no difference between the survival of males exposed to rivals or not. There was lower post‐mating survival overall for males held on the 0% yeast: 20% sugar and the agar‐only diets. The differences in post‐mating starvation survival did not match the pattern of extended matings between males exposed to rivals or held alone. This suggests that there is no straightforward relationship between future survival probability and adaptive responses to rivals.

We also tested to see whether significant differences observed in mating duration mapped on to the number of progeny produced from those matings. Previous work has shown that extended matings induce significantly higher fecundity and offspring production (Bretman *et al*., 2009), indicating that such responses are adaptive. However, here we found no elevated progeny production associated with any of the treatments exhibiting significantly extended matings. One potential explanation is that the mating tests were conducted in these experiments on agar‐only food medium, to prevent short‐term diet‐induced variation in the males’ reproductive strategies during the mating tests. Even though the females from these tests were then placed on normal medium to collect progeny, the exposure to agar only during the preceding mating tests may have precluded the observation of the expected progeny differences.

The study fits into a wider context of nutritional studies on reproductive traits and survival in both sexes of the focal species investigated here, and many such studies have used manipulations of yeast and sucrose components (e.g. Piper *et al*., 2005a, b; Partridge & Gems, 2006; Libert *et al*., 2007; Fricke *et al*., 2008, 2009; Grandison *et al*., 2009; Partridge *et al*., 2011; Wigby *et al*., 2011; Tatar *et al*., 2014). In high concentrations, dietary yeast has been found to be detrimental to female fecundity and lifespan (Bass *et al*., 2007). Dietary restriction (DR) has been shown to result in increased longevity but reduced fecundity (e.g. Chapman & Partridge, 1996; Chippindale *et al*., 1997) and the ability to respond to signals transferred during mating (e.g. Fricke *et al*., 2009). Hence, DR has a strong effect on overall female reproductive success.

The efficacy of male *D. melanogaster* to elicit post‐mating responses in females is also diet‐dependent (e.g. Fricke *et al*., 2008). This is important because in many species in which females mate multiply, as in *D. melanogaster*, a male's ability to succeed in sperm competition, which is aided significantly by the induction of female post‐mating responses, is of crucial importance. Examples of traits selected in this context include nuptial feeding and sperm partitioning between different females. However, a male whose food source is restricted may have limited options in terms of energy to invest in copulation, sperm competition or offspring production (Bass *et al*., 2007; Abraham *et al*., 2015). Responding to rivals in general will have costs (Bretman *et al*., 2013a) and males that have no resources will be less able to carry those costs, even if they could potentially respond adaptively. The results of our investigations here provide a new facet of the interaction of nutrition with reproductive success in showing that that diet can affect the ability of males to respond to rivals in a sexually competitive context.

This study also fits into a wider context of research into the effect of diet components on reproductive traits (e.g. Perez‐Staples *et al*., 2008, 2011; Taylor *et al*., 2013). For example, adult Tephritid fruit flies require continual carbohydrates and water to promote survival, and a protein source is required to attain sexual maturity (Aluja *et al*., 2001), maintain mating latency and mating frequency (Blay & Yuval, 1997). Similarly, males of the calliphorid fly (*Phormia regina*) have high reproductive success when fed high levels of protein in comparison with protein‐deprived males (Stoffolano *et al*., 1995). Dietary protein is also reported to affect male attractiveness/pheromone signalling in these fruit flies (e.g. Shelly *et al*., 2002). Prey deprivation/inadequate diets can lead to a partial or complete cessation of mating activity (Anderson & Franks, 2001; Perez‐Staples *et al*., 2008). For example, in the Mormon cricket *Anabrus simplex,* the number of sexually active males decreases when males are held on a nutritionally poor diet (Gwynne, 1993). Food intake also affects the size of reproductive structures and is positively related to testis size and mating duration in yellow dung flies (*Scatophaga stercoraria)* (Ward & Simmons, 1991). The finding of significant gene by nutritional environment interactions for male reproductive traits in *Tribolium castaneum* beetles (Lewis *et al*., 2012) interestingly suggests a role for diet in the maintenance of genetic variation in male reproductive success.

In future work, it will be interesting to determine whether the transfer of seminal fluid varies under a range of different diets. It would also be interesting to determine the longevity, fecundity and fertility of males held upon these different food treatments across sequential matings in order to test whether the differential manipulation of dietary components affects serial male allocation strategies. Further studies are also required on the specific ratios of diet components that can elicit adaptive responses among males and to further probe the underlying mechanisms involved.

## Conflict of interests

The authors declare that they have no conflict of interests.

## Supporting information


**Figure S1** Kaplan–Meier survival curves for males held on the six different diets. Dashed lines indicate males housed without rivals (100 vials per diet at one male per vial) and continuous lines indicate males housed in groups of four, that is with ‘rivals’ (100 vials per diet at four males per vial). In the ‘rivals’ treatments, dead males were removed daily and numbers per vial were kept constant by consolidating survivors.

**Figure S2** Post‐mating fitness and survival outcomes for males from Experiment 4. (a) Boxplots of 24‐h offspring production by females mated to males in the various treatments. Final sample sizes for ‘no rivals’, ‘rivals’ males were 100% Y: 100% S = 59, 60; 100% Y: 0% S = 59, 55; 0% Y: 100% S = 56, 57; 20% Y: 0% S = 54, 57; 0% Y: 20% S = 45, 51; 0% Y: 0% S = 54, 57. (b) Boxplots of post‐mating starvation resistance for mated males (days survived on agar‐only medium). Final sample sizes for ‘no rivals’, ‘rivals’ males were 100% Y: 100% S = 56, 60; 100% Y: 0% S = 59, 55; 0% Y: 100% S = 55, 56; 20% Y: 0% S = 55, 57; 0% Y: 20% S = 46, 53; 0% Y: 0% S = 54, 55. Significant planned contrasts: *P <* *0.05, **P <* *0.01, ***P <* *0.001.
