# Supplementary figures and images for: Resource limitation and responses to rivals in males of the fruit fly Drosophila melanogaster
Source: J Evol Biol. 2016 Jul 15;29(10):2010–21. doi: 10.1111/jeb.12924 (PMC5082519; doi:10.1111/jeb.12924)

Survival Probability

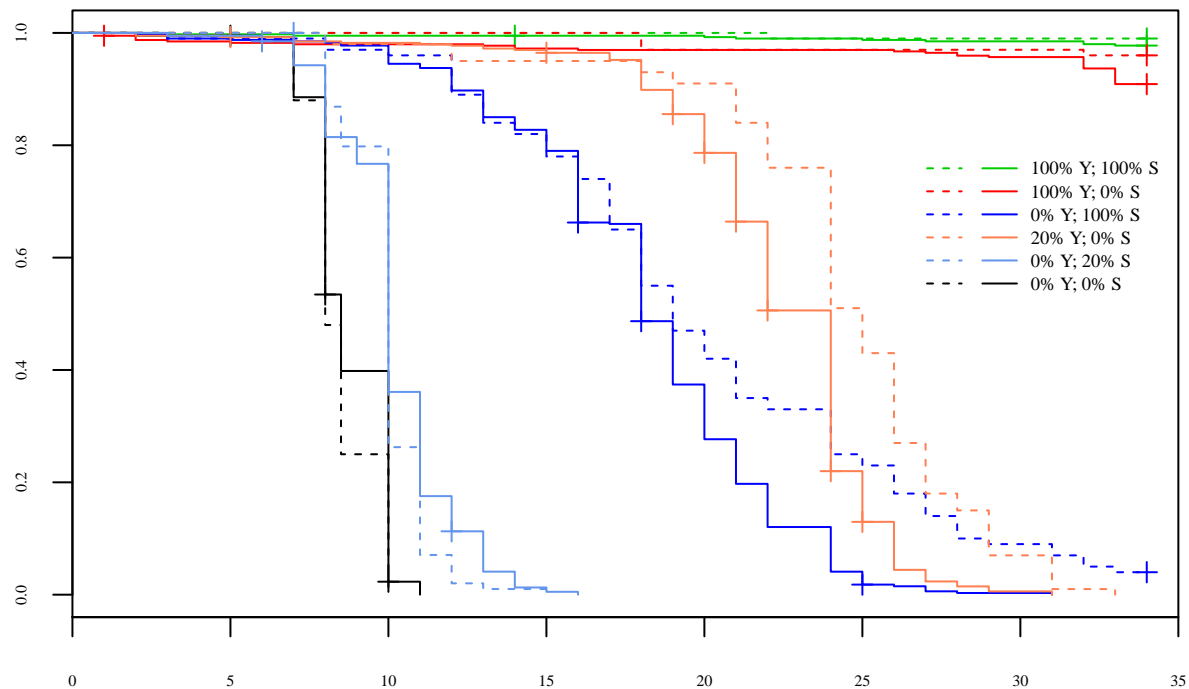

Age (days)

Supplement: Supplementary file 1 — Figure S1 Kaplan–Meier survival curves for males held on the six different diets. Dashed lines indicate males housed without rivals (100 vials per diet at one male per vial) and continuous lines indicate males housed in groups of four, that is with ‘rivals’ (100 vials per diet at four males per vial). In the ‘rivals’ treatments, dead males were removed daily and numbers per vial were kept constant by consolidating survivors. [file JEB-29-2010-s001.pdf]

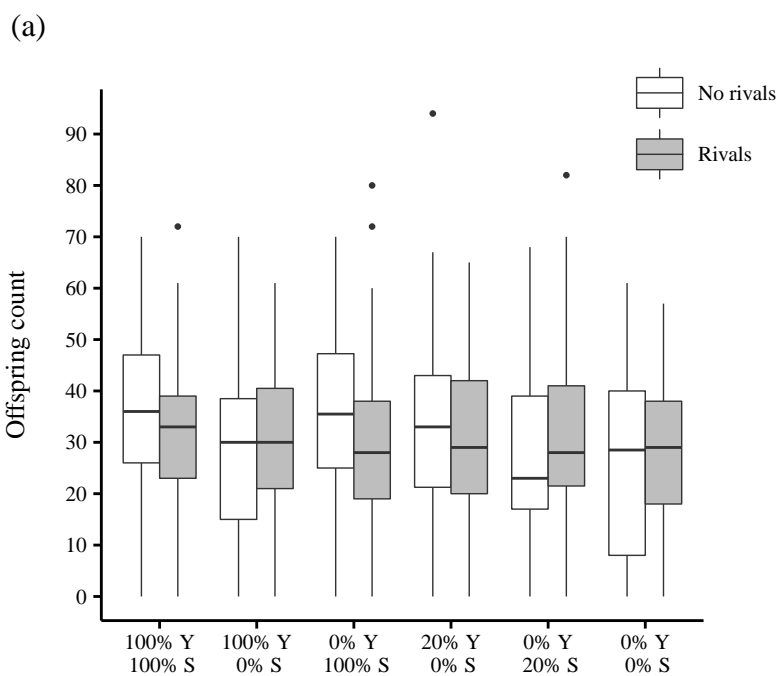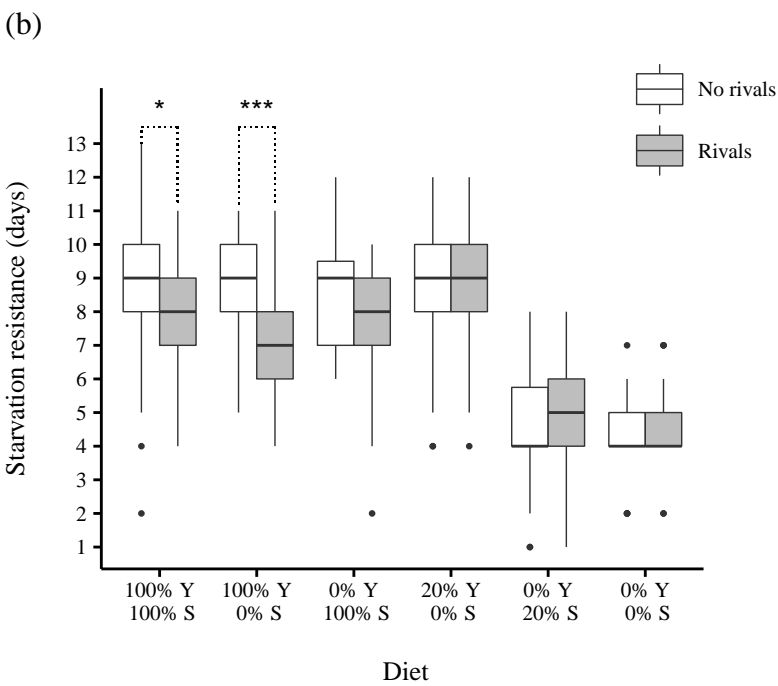

Supplement: Supplementary file 2 — Figure S2 Post‐mating fitness and survival outcomes for males from Experiment 4. (a) Boxplots of 24‐h offspring production by females mated to males in the various treatments. Final sample sizes for ‘no rivals’, ‘rivals’ males were 100% Y: 100% S = 59, 60; 100% Y: 0% S = 59, 55; 0% Y: 100% S = 56, 57; 20% Y: 0% S = 54, 57; 0% Y: 20% S = 45, 51; 0% Y: 0% S = 54, 57. (b) Boxplots of post‐mating starvation resistance for mated males (days survived on agar‐only medium). Final sample sizes for ‘no rivals’, ‘rivals’ males were 100% Y: 100% S = 56, 60; 100% Y: 0% S = 59, 55; 0% Y: 100% S = 55, 56; 20% Y: 0% S = 55, 57; 0% Y: 20% S = 46, 53; 0% Y: 0% S = 54, 55. Significant planned contrasts: *P < 0.05, **P < 0.01, ***P < 0.001. [file JEB-29-2010-s002.pdf]
